# Renal tubular Bim mediates the tubule-podocyte crosstalk via NFAT2 to induce podocyte cytoskeletal dysfunction

**DOI:** 10.7150/thno.43145

**Published:** 2020-05-22

**Authors:** Chunmei Xu, Xiaojun Zhou, Tianyue Xie, Yuan Zhou, Qian Zhang, Shan Jiang, Rui Zhang, Lin Liao, Jianjun Dong

**Affiliations:** 1 Department of Endocrinology and Metabology, Shandong Provincial Qianfoshan Hospital, Cheeloo College of Medicine, Shandong University, Jinan, Shandong, China.; 2 Laboratory of Endocrinology, Medical Research Center, the First Affiliated Hospital of Shandong First Medical University, Jinan, Shandong, China.; 3 Department of Endocrinology and Metabology, the First Affiliated Hospital of Shandong First Medical University, Jinan, Shandong, China.; 4 First Clinical Medical College, Shandong University of Traditional Chinese Medicine, Jinan, Shandong, China.; 5 Department of Endocrinology, Affiliated Hospital of Shandong University of Traditional Chinese Medicine, Jinan, Shandong, China.; 6 Department of Endocrinology, Qilu Hospital, Cheeloo College of Medicine, Shandong University, Jinan, Shandong, China.

**Keywords:** diabetic nephropathy, communication, cytoskeleton, Bim, coculture

## Abstract

Diabetic nephropathy (DN) is mainly regarded as diabetic glomerulopathy, and its progression is tightly correlated with tubular epithelial lesions. However, the underlying molecular mechanisms linking tubular damage and glomerulopathy are poorly understood.

**Methods:** We previously reported that the upregulation of Bim mediated proximal tubular epithelial cell (PTEC) apoptosis and was crucial in the early stages of DN. Herein we modulated Bim expression in PTECs and subsequently determined podocyte (PC) cytoskeletal arrangement by building a Transwell co-culture system in high glucose (HG).

**Results:** Compared to normal glucose, exposure to 40 mM of HG for 48 h induced significant expression of Bim in PTECs and disorganization in the PC cytoskeleton. When cocultured with PTECs in HG, exacerbated filamentous actin (F-actin) rearrangement and reduced synaptopodin levels were detected in PCs. In contrast, gene knockdown of Bim in PTECs was correlated with the absence of PC cytoskeletal disorganization. NFAT2 level and its nuclear translocation in PTECs were decreased by suppressing Bim expression. Upregulating NFAT2 disrupted the beneficial effects on F-actin organization in PCs obtained by inhibiting Bim. LncRNA microarray analysis identified NONHSAT179542.1, which was implicated in Bim-mediated PC cytoskeletal disorder.

**Conclusion:** Our study clarified the functional role of Bim, a pro-apoptotic factor, which is involved in the crosstalk between PTECs and PCs. Bim promotes NFAT2 activation in PTECs, inducing the downregulation of lncRNA NONHSAT179542.1 in PCs, contributing to the cytoskeletal damage. Identification of the role of the Bim/NFAT2 pathway may represent a promising research direction for a better understanding of DN development.

## Introduction

Diabetes mellitus (DM) is a growing public health problem worldwide with a high risk of severe complications 1-3. Diabetic nephropathy (DN) is one of the most serious and prevalent complications of DM and a major contributing factor to end-stage renal failure in up to 30% diabetics 4-8. Current recommendations of full renin-angiotensin system blockade 9, intensive insulin therapy, and stringent lipid and blood pressure control 10-12 mainly focus on mitigating the symptoms and retarding the progression to DN, but lack efficacy 13, with the residual risk of developing DN ranging between 46% to 84% 10,14. There is an urgent need to develop a more effective strategy for early control of DN and prevent further progression.

Accumulating evidence has indicated that tubular damage is a key cause of chronic kidney injury 15-17, which tightly correlates with the progression of DN and is superior to glomerular injury as a predictor of DN progression 18-21. Our previous research has shown that proximal tubular epithelial cell (PTEC) apoptosis in HG participated in the early DN development, while the podocyte (PC) damage was rarely observed 22, suggesting that tubular metabolic changes may precede changes in PCs in the early development of DN. Furthermore, the B cell lymphoma 2-interacting (Bcl2-interacting) mediator, Bim, was identified to play a critical role in PTECs' apoptosis in HG 22. Bim is constitutively expressed in many cell types 23, and is primarily required for the initiation of apoptosis provoked by various stress stimuli 24,25. Bim was also reported to increase cytokine production 26, which provided strong evidence that intracellular Bim could influence peripheral cells.

The glomerulus is centrally involved in the development of DN 27,28. The main manifestations of DN include albuminuria and damage to the glomerular filtration barrier (GFB), followed by proteinuria contributing to its progression. The molecular mechanisms underlying the onset of early lesions with tubular apoptosis leading to the progression of DN are not fully elucidated. We have previously demonstrated that Bim mediated tubular apoptosis 22. Whether Bim affects peripheral PCs or mediates crosstalk between PTECs and PCs remains unknown.

In this study, we elucidated the functional role of the Bim/NFAT2 pathway in the crosstalk between renal tubules and PCs that was involved in DN development. The early dysfunction of PCs in DN was preliminarily caused by actin remodeling abnormalities and cytoskeletal rearrangement 29,30. Specifically, we observed that when PCs were cocultured with PTECs in HG, there was increased expression of Bim in PTECs in response to HG. Elevated expression of Bim then mediated the activation of downstream target NFAT2, rendering PCs susceptible to cytoskeletal damage. Additionally, lncRNA microarray analysis revealed that lncRNA NONHSAT179542.1 was involved in PCs' cytoskeletal reorganization. In summary, by constructing the* in vitro* co-culture system to emulate *in vivo* environment, we have identified the underlying molecular mechanism of the crosstalk between PCs and PTECs during the early stage of DN pathogenesis.

## Materials and Methods

### Culture of PCs and PTECs

Conditionally immortalized human PCs were donated by Dr. Yi Fan (Department of Pharmacology, Shandong University School of Medicine, Jinan, China) and cultured in RPMI-1640 medium (Corning, NY, USA) supplemented with 10% fetal bovine serum (FBS) (Lonsera, Uruguay), 100 units/mL penicillin and 100 mg/mL streptomycin (#15140-122, Gibco, USA) until the cells became confluent. HK-2, a human PTEC line which had been confirmed by short tandem repeat (STR) profiling, was purchased from ProCell Corporation (Wuhan, China) and maintained in RPMI-1640 medium with 10% FBS, 100 units/mL penicillin and 100 mg/mL streptomycin. All cells were grown at 37 °C with 5% CO_2_ and were passaged every other day.

### Determination of high glucose (HG) concentration

To determine the optimal concentration of treatment with HG and treatment time, PCs were cultured in 6-well plates for 24 h, 48 h, and 72 h in RPMI-1640 medium containing 5.5 mM glucose supplemented with 0, 10, 20, 30, 40, or 50 mM of D-glucose (#G8270, Sigma, Oakville, ON, Canada). Subsequently, PCs were collected for detecting the level of the cytoskeleton-related protein, synaptopodin. Mannitol was used to achieve equivalent osmotic pressure, and L-glucose (#G5500, Sigma, Oakville, ON, Canada, a nonmetabolizable isomer of glucose) served as a control to determine the uniqueness of D-glucose.

### Transwell co-culture system construction and Grouping

Transwell cell-culture inserts (Catalogue: 3450, pore size: 0.4 μm; Corning Costar Corp., NY, USA) were placed in RPMI-1640 medium with 10% FBS and 1% antibiotics in the upper and lower compartments. The heights of the medium in the upper and lower compartments were maintained at similar levels, so the bulk flow was not due to a hydrostatic pressure gradient. PCs were resuspended at 5 ×10^4^/well in the lower chamber (a 6-well plate), which contained 40 mM HG medium; the upper chambers containing the HG medium were seeded with 5 × 10^4^/mL control PTECs or PTECs transfected with Lenti-virus (Lenti-Bim-shRNA) as described in the “Bim Lentiviral vector construction” section below. There was communication between different cells in the system through a Polyester (PET) membrane for 24 h, 48 h, and 72 h. As controls, PCs were also cultured in 6-well plates under normal glucose (NG) and HG medium without inserts.

The experimental groups included: monoculture of PCs in NG and HG, coculture of PCs with control PTECs in HG, coculture of PCs with PTECs transfected with Lenti-Bim-shRNA (PTEC-Bim-shRNA) in HG, and coculture of PCs with PTECs transfected with negative control virus (PTEC-Bim-shNC) in HG. All co-cultures were set up in triplicate.

### Treatment of PTECs in the upper chamber

#### Bim Lentiviral vector construction

Bim Lentiviral vector was purchased from GeneChem Corporation (Shanghai, China). Bim short hairpin RNA (shRNA) target sequence, CAC AGT TCG AGC GAT CTG TTA, was cloned into the hU6-MCS-Ubiquitin-EGFP-IRES-puromycin vector (Lenti-Bim-shRNA). The same vector was used to insert the sequence TTC TCC GAA CGT GTC ACG T as a negative control (Lenti-Bim-shNC). PTECs at 30-40% confluency were transfected with Lenti-Bim-shRNA (PTEC-Bim-shRNA) or Lenti-Bim-shNC (PTEC-Bim-shNC) according to the manufacturer's protocol (GeneChem, Shanghai, China). After 48 h transfection, the cells were selected with 1 μg/mL puromycin for one week. The PTEC lines with stable knockdown of Bim were established. RNA was extracted, and lysates were collected for Western blotting to confirm the efficiency of the intervention.

#### Screening and validation of downstream target of Bim

The RNA of PTECs from different upper chambers of the Transwell co-culture system was extracted, including control PTECs, PTEC-Bim-shRNA, and PTEC-Bim-shNC. The RNA was reverse-transcribed into cDNA, which was further amplified. Quantitative real-time polymerase chain reaction (RT-PCR) was performed to detect the mRNA expression level of NFAT family members, including NFATc1, NFATc2, NFATc3, NFATc4, and NFATc5, following alteration by Bim. The subtype of NFAT, which was regulated by Bim, was determined, and Western blotting was conducted to confirm the relationship between Bim and the downstream target effector.

#### Confocal microscopy for NFAT2 translocation

PTEC-Bim-shRNA and PTEC-Bim-shNC as well as un-transfected controls were fixed in 4% paraformaldehyde for 30 min followed by permeabilization in 0.5% Triton X-100 for 5 min. Subsequently, cells were incubated with primary antibodies against NFAT2 (1:100, ab25916, Abcam) at 4 °C overnight. After washing, the slides were stained with Alexa Fluor 594-conjugated secondary antibodies (1:50, Invitrogen, CA, USA) and double-stained with Hoechst 33342 (Thermo Fisher Scientific, Waltham, MA, USA) to visualize nuclei. Slides were rinsed twice with PBS and mounted on glass coverslips using ProLong™ Glass Antifade Mountant (Thermo Fisher Scientific, Waltham, MA, USA). Cells were observed, and images were captured at randomly selected fields using an LSM780 confocal microscope (Carl Zeiss, Jena, Germany).

#### Downregulation and overexpression of NFATc1

The PTEC-Bim-shRNA were transiently transfected with NFATc1 (GeneChem, Shanghai, China) or control empty plasmids. Briefly, Opti-MEM medium (Gibco-BRL/Invitrogen, Carlsbad, CA), containing 2.5 μg p-NFATc1 or empty plasmids, was mixed with 5 μL lipofectamine 3000 transfection reagent (Thermo Fisher Scientific, Waltham, MA, USA) and the mixture was added to the cells. After 8-12 h, the Opti-MEM medium was removed, and the cells were incubated with RPMI-1640 medium containing 10% FBS. Forty-eight hours after the transfection, plasmid transfection efficiency was evaluated by RT-PCR and Western blotting.

The siRNAs against NFATc1 were transfected into control PTECs using lipofectamine 2000 transfection reagent (Thermo Fisher Scientific, Waltham, MA, USA) by following the manufacturer's instructions. The siRNAs against NFATc1 (siNFATc1) were synthesized by Shanghai GenePharma. Sense sequences for NFATc1 siRNAs were 5'-GCA UGG CUA CUU GGA GAA UTT-3' (siNFATc1-1) and 5'-AUU CUC CAA GUA GCC AUG CTT-3' (siNFATc1-2). Sense sequences for the negative control were 5'-UUC UCC GAA CGU GUC ACG UTT-3' (siNC-1) and 5'-ACG UGA CAC GUU CGG AGA ATT-3' (siNC-2). The siRNA knockdown efficiency was verified by RT-PCR and Western blotting. Thus, PTECs with stable knockup (p-NFATc1) and knockdown of NFATc1 (siNFATc1) were established. Each transfection was repeated in triplicate. After successful transfection, the cells were seeded in the upper chamber of the Transwell system and were cocultured with PCs in HG condition by following the instructions described.

### Treatment of PCs in the lower chamber

#### Determination of cytoskeleton-related protein synaptopodin expression

After monoculture of PCs and coculturing with various PTECs in which Bim expression was regulated by various treatments in HG for specific periods (24 h, 48 h, 72 h), synaptopodin expression in PCs was detected as described in the “Immunofluorescence (IF) staining” and “Western blotting” section below.

After the downstream target of Bim in PTECs was verified, synaptopodin expression in various PCs was examined as follows: PC groups cocultured with PTECs carrying various vectors including, siNFATc1, siNC, Lenti-Bim-shRNA, Lenti-Bim-shRNA carrying p-NFATc1, and Lenti-Bim-shRNA carrying empty plasmid as well as control PTECs.

#### Immunofluorescence (IF) staining

PCs were seeded on sterile glass coverslips in the lower chamber of the Transwell system or a sole 6-well plate and cultured under various conditions. Subsequently, glass coverslips with PCs were extracted to detect IF intensity. The slides were fixed for 30 min with 4% paraformaldehyde, followed by permeabilization with 0.5% Triton X-100 in PBS for 5 min. Thereafter, the slides were incubated with the TRITC Phalloidin (1:200, CA1610, Solarbio, Beijing, China) or synaptopodin antibody (1:50, 21064-1-AP, Proteintech) at 4 °C overnight. Next day, Alexa Fluor 594-conjugated secondary antibodies (1:50, Invitrogen, CA, USA), along with DAPI stain to visualize the nuclei, were applied to detect synaptopodin. Images were taken at randomly selected fields using an OLYMPUS FSX100 imaging system (Olympus, Tokyo, Japan).

After the regulation of NFAT2, IF staining for synaptopodin and F-actin fiber arrangement in PCs was also performed in a similar manner as delineated above.

### Quantitative RT-PCR

Total RNA was extracted from the cultured cells using TaKaRa RNAiso Plus (Cat.#9108) following the manufacturer's protocol, and the concentration of the total RNA was quantified by measuring the absorbance at 260 nm. RNA from each sample (1 μg) was added to 20 μL reaction mixture, and cDNA was synthesized using the PrimeScript™ RT reagent Kit with genomic DNA Eraser (Takara, Cat# RR047A). Quantitative RT-PCR was performed using UltraSYBR Mixture (low ROX) (CWBIO, Inc., Beijing, China) to detect Bim, NFATc1, NFATc2, NFATc3, NFATc4 and NFATc5 mRNA expression. β-actin was used as an internal control. The primer sequences are shown in Table S1. The 2^-ΔΔCT^ method was used to calculate the relative expression levels of each mRNA. RT-PCR was performed in triplicate.

### Western blotting

Proteins were isolated from various cultured PTECs and PCs, and protein levels were detected. Briefly, protein extracts were boiled in RIPA buffer (Beyotime, Shanghai, China) and separated by SDS-PAGE electrophoresis. Western blotting was performed with the antibodies against synaptopodin (syn) (1:1000, ABN481, Millipore) to detect its protein level in PCs. Anti-Bim antibodies (1:1000, ab32158, Abcam) were used to examine the HG-induced Bim expression and the inhibition effect on PTECs after transfection with the Lentiviral vector. Antibodies against NFAT2 (1:1000, ab25916, Abcam) were used to detect NFAT2 levels after transfection with siNFATc1 or p-NFATc1 into PTECs or PTEC-Bim-shRNA. Anti-MICAL2 antibodies (1:1000, 13965-1-AP, Proteintech) were used to detect the MICAL2 level in PCs after coculturing with un-transfected PTECs and PTEC-Bim-shRNA, and after transfection of NONHSAT179542.1 siRNA and siNC into PCs. β-actin (1:5000, 60008-1-Ig, Proteintech) was used as a reference protein.

### Profiling of long noncoding RNA (lncRNA) expression and identification of differential lncRNAs

Total RNA was isolated using TaKaRa RNAiso Plus (Cat. #9108) according to the manufacturer's instructions. Total RNA was assessed for a RIN number using Agilent Bioanalyzer 2100 (Agilent Technologies, Santa Clara, CA, US). The biotinylated cRNA targets for the Sino-Human lncRNA array V3.0. were generated by RNA samples of each group and then were hybridized with probe on the slides. Upon hybridization, slides were scanned on the Agilent Microarray Scanner (Agilent Technologies, Santa Clara, CA, US). Data extraction was conducted with Feature Extraction software 10.7 (Agilent Technologies, Santa Clara, CA, US). The Quantile algorithm of R package “limma” was used to normalize raw data. The microarray experiments and data analysis were performed by Shanghai Sinomics Corporation (Shanghai, China) following the protocol of Agilent technologies Inc at Sinotech Genomics Corporation. The lncRNAs were constructed using the public transcriptome databases (e.g. NCBI, Ensembl, NONCODE, and Lncipedia). The microarray also included an entire collection of 25353 protein-coding mRNAs. Genes with a fold change of at least 1.5 were selected for further analysis. Heatmap was generated by an R package “pheatmap”.

### Functional annotation and Target gene prediction of lncRNAs

Kyoto Encyclopedia of Genes and Genomes (KEGG) pathway enrichment analysis was performed to identify significant pathways. Target gene prediction of lncRNAs by cis/trans was conducted. Cis target gene prediction was to seek target mRNA located near lncRNA within 10 kb. Trans target gene prediction was carried out following the principle of sequence complementary pairing. Complementary mRNA with lncRNA was acquired by blast alignment. We used RNAplex software to calculate the thermodynamic parameters of lncRNA complementary with mRNA, and the sequence e ≤30 was extracted. The target network was constructed using Cytoscape.

### Validation of lncRNAs by quantitative RT-PCR and regulation by transfected with siRNA

Expression of fourteen lncRNAs was validated by RT-PCR. The cDNA was synthesized by using a PrimeScript™ RT reagent kit (TaKaRa). Then, RT-PCR was performed using UltraSYBR Mixture (low ROX) (CWBIO, Inc., China). Primers for NONHSAT253617.1, XR_929282.2, NONHSAT179542.1, NONHSAT251382.2, XR_934942.2, NONHSAT220341.1, ENST00000542022, NONHSAT040129.2, NONHSAT227535.1, NONHSAT203700.1, NONHSAT243847.1, NONHSAT040287.2, NONHSAT179858.1, and NONHSAT179237.1 were synthesized by RiBo-Bio (Guangzhou, China). All RT-PCR primer sequences are shown in Table S1. β-actin was used as an internal control.

After validation, the sequence of siRNA against NONHSAT179542.1 was designed by RiBo-Bio (Guangzhou, China). The sequence of siRNA against NONHSAT179542.1 was as follows: 5'- ACTTGGAAACTGCCAGAAA-3'. PCs after coculturing with PTEC-Bim-shRNA were transfected with 50 nM NONHSAT179542.1 siRNA or siNC using Lipofectamine™ 2000 (Invitrogen) according to the manufacturer's protocols. After transfection for 48 h, the RNA was extracted and the intervention effect was validated by RT-PCR.

### Cytoskeletal organization and synaptopodin expression following regulation of NONHSAT179542.1

To determine the influence of NONHSAT179542.1 on the PC cytoskeleton organization, IF staining for F-actin and synaptopodin was conducted. Synaptopodin expression in PCs after downregulation of NONHSAT179542.1 was examined by Western blotting.

The groups involved cocultures of control PTECs, PTEC-Bim-shRNA, and PTEC-Bim-shNC with PCs. Also, PTEC-Bim-shRNA were cocultured with PCs transfected with NONHSAT179542.1 siRNA (PTEC-Bim-shRNA + PC-lncRNA-siRNA), or with NONHSAT179542.1 siNC (PTEC-Bim-shRNA + PC-lncRNA-siNC). All co-cultures were set up in triplicate.

### Statistical analysis

All statistical analyses were performed using SPSS Statistics 22.0 (SPSS Inc, Chicago, USA). A Student's t-test was used to assess significance for data within two groups. Multiple statistic comparisons were analyzed using one-way ANOVA, followed by post hoc tests. Data were presented as mean ± standard error of the mean (S.E.M.), and the level of statistical significance was estimated at *P* < 0.05.

## Results

### HG induced disordered cytoskeletal arrangement in cultured human PCs

The specific effect of HG on the cytoskeletal arrangement was determined by synaptopodin expression, which was proportional to the number of physical cytoskeletal filaments. As shown in Figure S1, compared with the normal control, 40 mM D-glucose exposure for 48 h significantly reduced the abundance of synaptopodin (*P* < 0.05); other glucose concentrations showed no significant effect. HG treatment of D-glucose for 24 h and 72 h had no significant difference in the synaptopodin level compared with the normal group (Figure S1). PCs treated with high L-glucose exhibited higher synaptopodin expression than D-glucose treatment, demonstrating the unique effect of D-glucose on the cytoskeletal disorganization (Figure S2).

### Bim expression in PTECs was increased in response to HG

**Figure 1:**
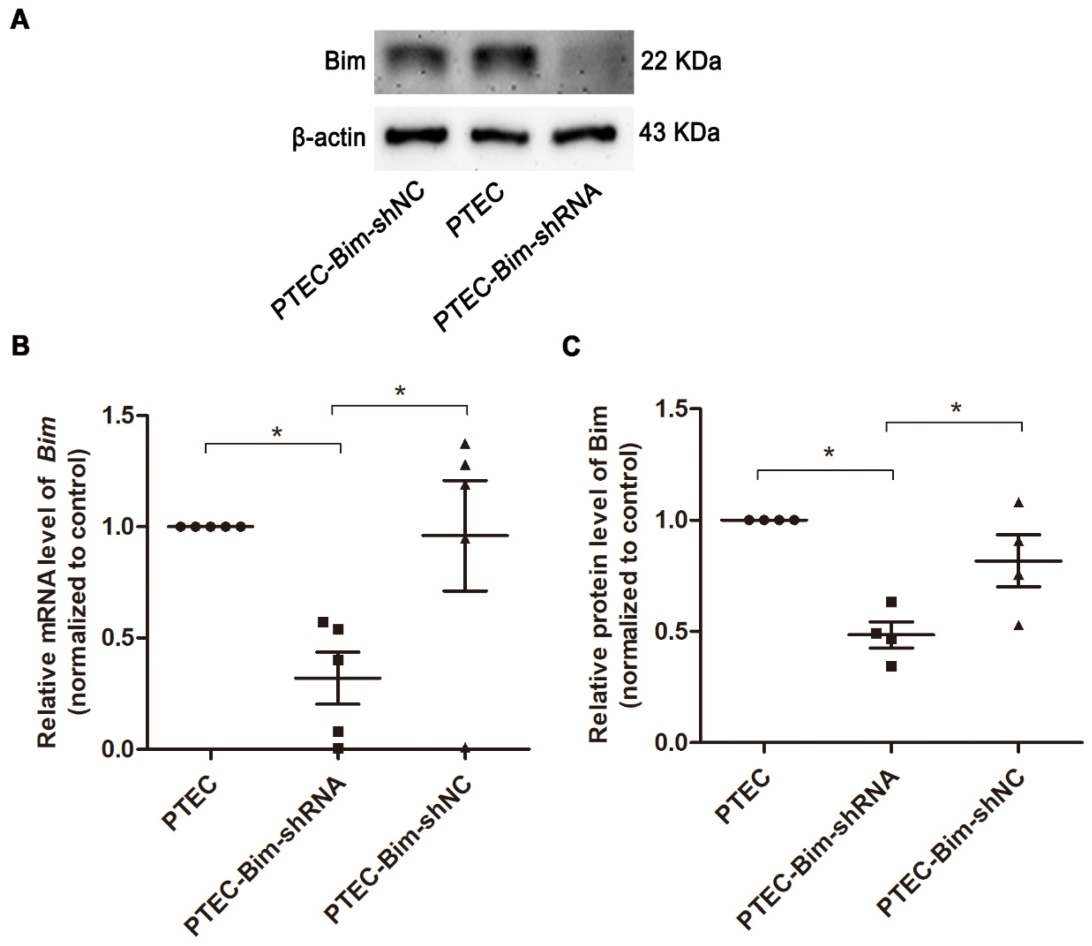
** Analysis of Bim expression in PTECs after transfection with various Lenti-virus constructs.** (**A**) Western blotting showed significant downregulation of Bim in PTECs when transfected with Lenti-virus (Lenti-Bim-shRNA) compared to transfection with negative control Lenti-virus (Lenti-Bim-shNC). (**B**) Bim mRNA in PTECs was detected by RT-PCR. (**C**) Quantification of Bim protein expression in PTECs was detected by Western blotting. Data are mean ± S.E.M. *P < 0.05. PTECs: proximal tubular epithelial cells. PTEC-Bim-shRNA: PTEC transfected with Lenti-Bim-shRNA; PTEC-Bim-shNC: PTEC transfected with Lenti-Bim-shNC.

As shown in Figure S3, the protein expression of Bim was significantly increased in PTECs induced by 40 mM HG compared to normal controls (*P* < 0.05). To investigate the role of increased Bim in PCs cytoskeletal organization, Bim expression was further regulated. The expressions of Bim in PTECs after alteration were investigated by RT-PCR and Western blotting analysis. Compared with the vehicle control, Bim expression was significantly decreased in cultured PTECs (*P* < 0.05) following transfection with Lenti-Bim-shRNA (Figure 1).

### Loss of Bim in PTECs alleviated cytoskeletal disarrangement and increased synaptopodin expression in PCs

**Figure 2:**
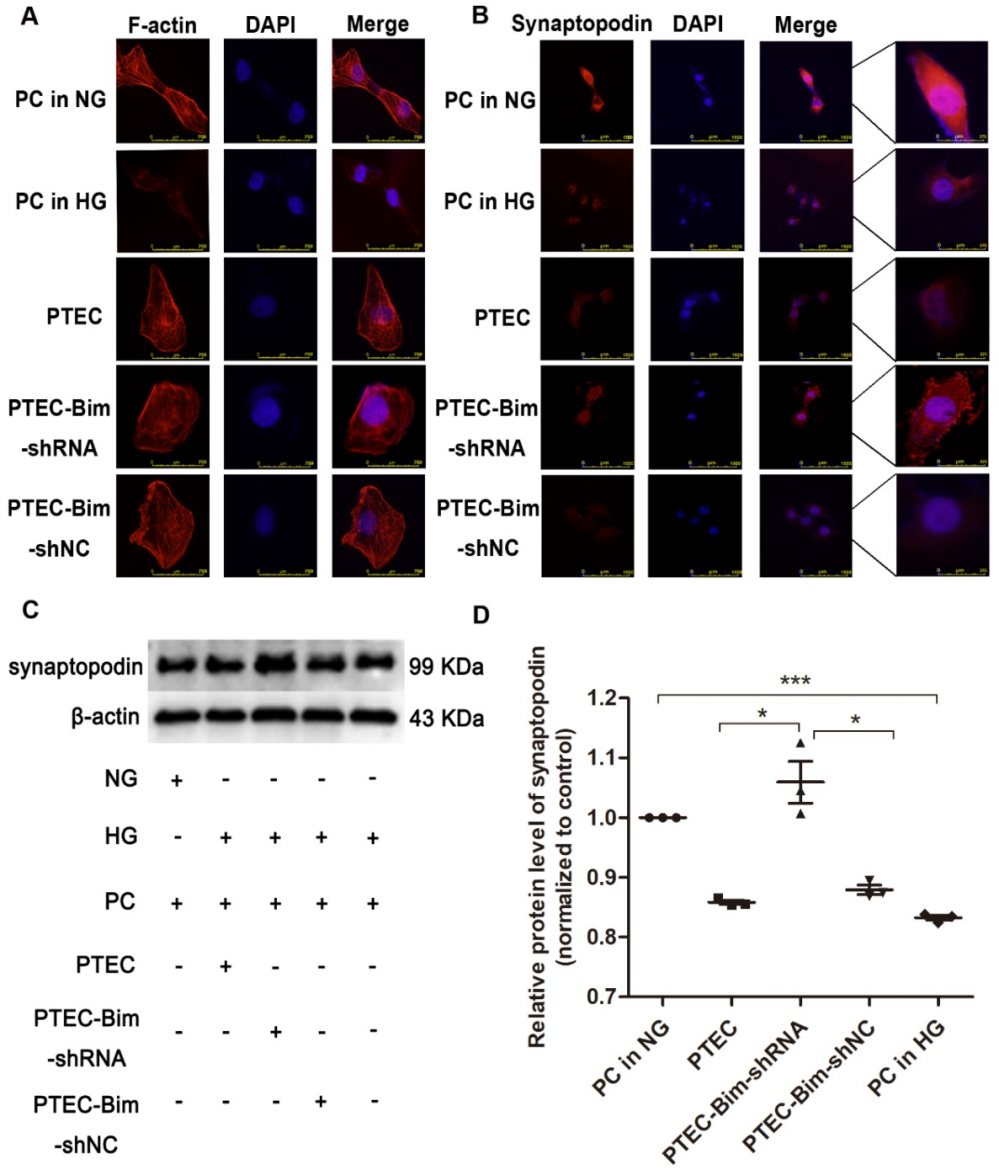
** Effect of Bim in PTECs on podocyte (PC) cytoskeletal organization detected by IF staining and Western blotting.** (**A**) IF staining showed orderly orchestrated F-actin fibers in PCs of the NG group and disordered cytoskeleton in PCs treated with HG. Suppression of Bim in PTECs markedly ameliorated the cytoskeletal disorganization in PCs by coculturing with PTECs in HG. Original magnification: 400×. (**B**) IF staining showed lower expression of synaptopodin in PCs in response to HG compared with NG. Suppression of Bim in PTECs significantly upregulated synaptopodin level in PCs. Original magnification: 200×; the magnified image is shown in the right panel of each picture. (**C**) Significant decrease in synaptopodin level in HG compared with NG by Western blotting. Synaptopodin level was significantly up-regulated in PCs after coculturing with PTEC-Bim-shRNA to inhibit Bim expression compared to those cocultured with the PTEC-Bim-shNC. (**D**) Quantification of synaptopodin protein expression in PCs was detected by Western blotting. Data are the mean ± S.E.M. *P < 0.05. NG: normal glucose. HG: high glucose. PC: podocyte.

We analyzed F-actin organization and synaptopodin expression in PCs when Bim levels were decreased. As shown in Figure 2A, there were numerous blue dots and regular red fibers, which were often overlaid in the merged images in cells cultured in 5.5 mM glucose medium, illustrating a baseline cytoskeleton arrangement with orderly phalloidin-stained F-actin fibers. On the contrary, HG exposure for 48 h markedly reduced the regular red fibers but not the blue dots in the merged image, suggesting the damage of orderly phalloidin-stained F-actin fibers. When cocultured with PTECs in HG, a severely disordered structure of F-actin fibers in PCs was observed (Figure 2A). The disordered cytoskeleton was reversed when cocultured with PTEC-Bim-shRNA in HG for 48 h (Figure 2A).

Furthermore, IF staining showed that normal synaptopodin expression was present in PCs cultured in NG, whereas HG treatment for 48 h reduced the IF intensity of synaptopodin in PCs compared with the NG treatment (Figure 2B). When cocultured with PTECs, decreased synaptopodin expression in PCs was observed, but a significant improvement was achieved by inhibiting Bim expression in PTECs (Figure 2B). Western blotting showed that in contrast to NG, HG treatment for 48 h dramatically reduced the level of synaptopodin in PCs (*P* < 0.05, Figure 2C-D). Compared with PCs cocultured with control PTECs in HG for 48 h, decreased expression of synaptopodin in PCs was recovered when cocultured with PTEC-Bim-shRNA (*P* < 0.05), which inhibited Bim expression in PTECs (Figure 2C-D).

### Bim regulated NFAT2 expression and translocation in PTECs

**Figure 3:**
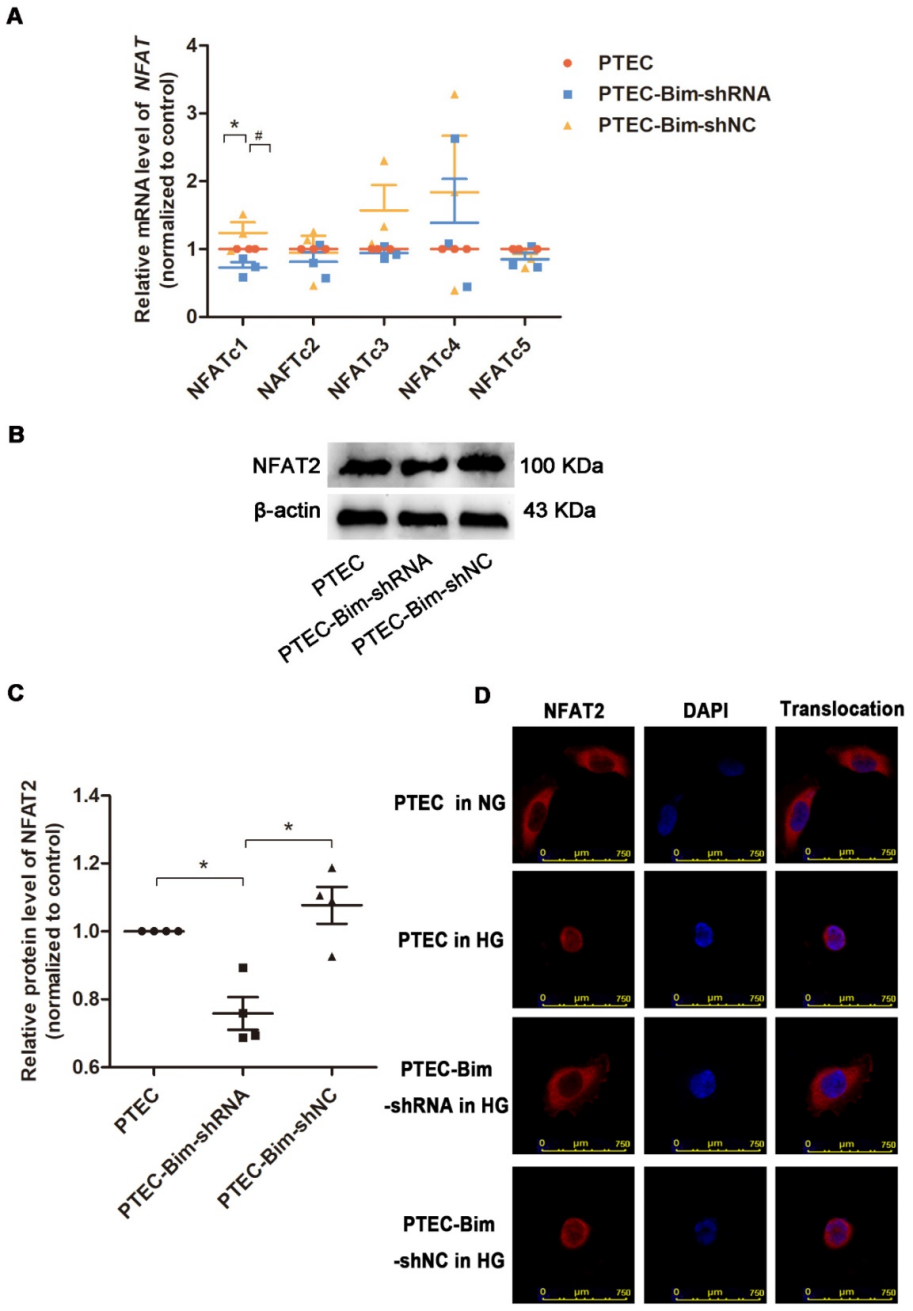
** Screening for the NFAT subtype regulated by Bim alteration and validation of NFAT2 expression and nuclear translocation.** (**A**) RT-PCR showed that NFATc1 was controlled by Bim alterations. *P < 0.05, PTECs versus PTEC-Bim-shRNA. ^#^*P* < 0.05. PTEC-Bim-shRNA versus PTEC-Bim-shNC. (**B**) Western blotting confirmed that NFAT2 was regulated by Bim and was significantly decreased by inhibiting Bim in PTECs. (**C**) Quantification of NFAT2 protein expression in PTECs after the regulation of Bim by Western blotting. *P < 0.05. (**D**) IF staining showed that NFAT2 nuclear translocation was triggered by HG stimulation, whereas suppression of Bim diminished HG-induced nuclear accumulation of NFAT2. Original magnification: 400×. Data are mean ± S.E.M.

We investigated the downstream target of Bim in PTECs. As shown in Figure 3A, RT-PCR analysis showed that when Bim expression was downregulated, NFATc1 mRNA level was decreased compared with control PTECs (*P* < 0.05). Results of Western blotting analysis were consistent with RT-PCR and NFAT2 expression was found to be regulated by Bim in PTECs (*P* < 0.05, Figure 3B-C).

For further verifying the regulation of NFAT2 function by Bim, the nuclear translocation of NFAT2 was examined. IF staining demonstrated that nuclear NFAT2 protein expression was up-regulated, whereas its cytoplasmic expression was markedly decreased in PTECs when treated with HG versus NG (Figure 3D), suggesting a markedly increased nuclear translocation of NFAT2 in response to HG. Conversely, the nuclear NFAT2 protein significantly declined and cytoplasmic expression enhanced in Bim-silenced PTECs, manifesting a reversed effect of Bim inhibition on the NFAT2 nuclear translocation (Figure 3D).

### Overexpression of NFAT2 in PTECs mediated PC cytoskeletal disarrangement

**Figure 4:**
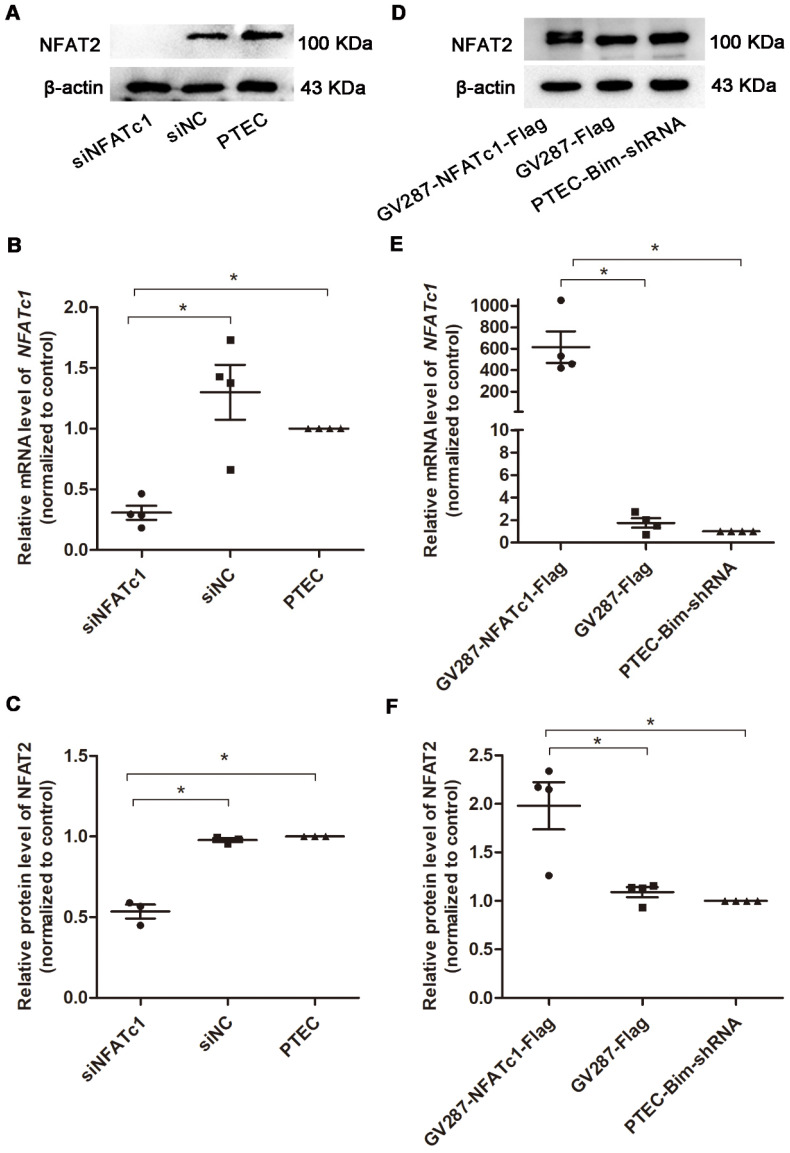
** Suppression and overexpression of NFAT2 in PTECs.** (**A**) Western blotting showed significant downregulation of NFAT2 in PTECs after transfection with NFATc1-siRNA. (**B**) Expression of NFATc1 mRNA in PTECs transfected with NFATc1-siRNA detected by RT-PCR. (**C**) Quantification of NFAT2 protein expression in PTECs transfected with NFATc1-siRNA detected by Western blotting. (**D**) Western blotting showed overexpression of NFAT2 in PTEC-Bim-shRNA after transfection with a plasmid carrying NFATc1. (**E**) Expression of NFATc1 mRNA in PTEC-Bim-shRNA transfected with a plasmid carrying NFATc1 detected by RT-PCR. (**F**) Quantification of NFAT2 protein expression in PTEC-Bim-shRNA transfected with a plasmid carrying NFATc1 detected by Western blotting. Data are mean ± S.E.M. *P < 0.05.

To confirm Bim-mediated PC cytoskeletal disarrangement via NFAT2, we regulated Bim and NFAT2 expression in PTECs and determined F-actin arrangement and synaptopodin level in PCs. RT-PCR and Western blotting analyses revealed that PTECs after transfection with siNFATc1 showed downregulation of NFAT2 compared with control PTECs (*P* < 0.05, Figure 4A-C). As shown in Figure 4D-F, compared with PTEC-Bim-shRNA cells, NFAT2 expression was increased when transfected with p-NFATc1 (*P* < 0.05).

**Figure 5:**
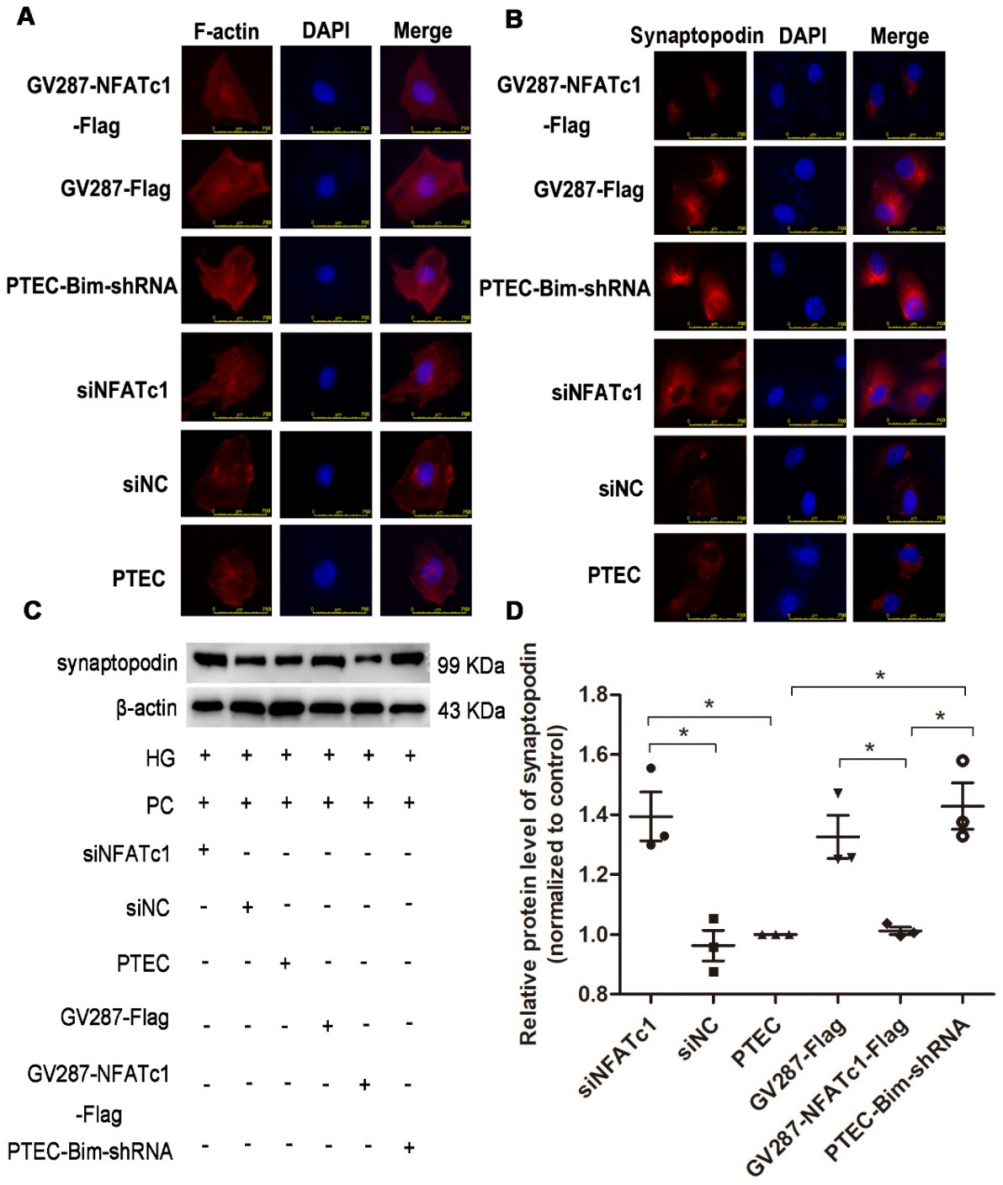
** Effect of Bim-induced NFAT2 alterations in PTECs on PC cytoskeletal organization.** (**A**) IF staining showed that suppression of NFAT2 markedly alleviated disorganized PC cytoskeleton induced by coculturing with PTECs in HG. On the contrary, upregulation of NFAT2 impaired ordered cytoskeletal organization induced by coculturing with PTEC-Bim-shRNA. Original magnification: 400×. (**B**) IF staining for synaptopodin showed that inhibition of NFAT2 resulted in its overexpression compared with the control, while increased synaptopodin level induced by suppression of Bim was disrupted by overexpression of NFAT2. Original magnification: 400×. (**C**) Western blotting demonstrated NFAT2 inhibition in PTECs increased synaptopodin expression in PCs, while its expression was decreased after transfection with NFAT2-plasmid into PTEC-Bim-shRNA. (**D**) Quantification of synaptopodin protein expression in PCs after regulation of NFAT2 in PTECs and PTEC-Bim-shRNA by Western blotting. Data are mean ± S.E.M. *P < 0.05.

IF staining for F-actin showed that suppression of NFAT2 induced improved arrangement of F-actin (Figure 5A), suggesting that NFAT2 was involved in the cytoskeletal disorder of PCs. An ordered arrangement of F-actin fibers was also obtained in PTEC-Bim-shRNA compared with control PTECs (Figure 5A). However, despite the knockdown of Bim in PTECs, overexpression of NFAT2 in PTEC-Bim-shRNA achieved inexorable damage in F-actin reorganization in PCs (Figure 5A), suggesting that NFAT2 is a potential target of Bim involved in the cytoskeleton damage of PCs.

Similar results were observed from the IF staining of synaptopodin (Figure 5B). Western blotting analysis revealed that compared with control PTECs, the level of synaptopodin in PCs was increased when cocultured with PTECs transfected with siNFATc1 (Figure 5C-D). Also, when cocultured with PTEC-Bim-shRNA, synaptopodin level in PCs was significantly increased compared with control PTECs, whereas overexpression of NFAT2 in PTEC-Bim-shRNA reversed the benefit achieved by inhibition of Bim (Figure 5C-D).

### Identification of differential lncRNAs

**Figure 6:**
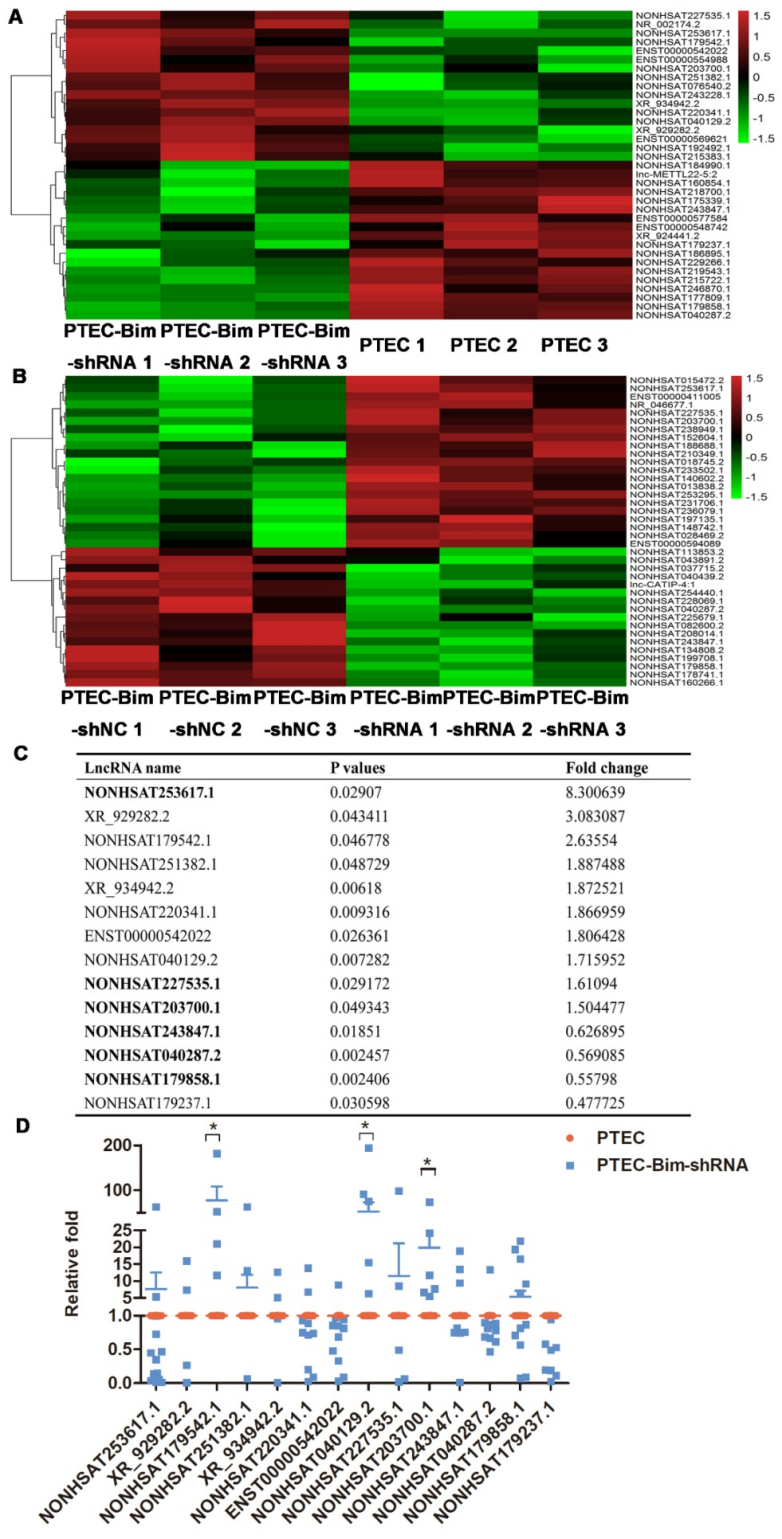
** Heatmap of lncRNA microarray data** (**A**) Differentially expressed lncRNAs between PTEC-Bim-shRNA and control PTECs (**B**) Differentially expressed lncRNAs between PTEC-Bim-shNC and PTEC-Bim-shRNA. Red points represent upregulated lncRNAs with statistical significance (fold change > 1.5,* P* < 0.05), while green points represent downregulated lncRNAs with statistical significance. (**C**) Fourteen lncRNAs were selected by choosing the top 10 differential lncRNAs in the PTEC-Bim-shRNA vs control PTECs and the 6 overlaps (bold) between PTEC-Bim-shRNA vs control PTECs and PTEC-Bim-shNC vs PTEC-Bim-shRNA. (**D**) Results of 14 lncRNAs expression were verified in PTEC-Bim-shRNA vs control PTECs by RT-PCR. NONHSAT179542.1 expression in PCs was markedly upregulated by inhibiting Bim expression in PTECs. Data are mean ± S.E.M. *P < 0.05.

To systematically identify lncRNAs in PCs after various treatments, we compared differential lncRNA expression profile by microarray analysis of PCs between the two cocultured groups (PTEC-Bim-shRNA vs control PTECs). A heatmap between two groups revealed differentially expressed lncRNAs with statistical significance (fold change > 1.5, *P* < 0.05). Variations of lncRNA expression from the lncRNA microarray data are shown in Figure 6A-B. Among 35 lncRNAs with differential expression, 17 lncRNAs were upregulated while 18 were downregulated in PCs cocultured with PTEC-Bim-shRNA compared with those cocultured with control PTECs. We also compared lncRNA microarray data from PCs cocultured with PTEC-Bim-shRNA and PTEC-Bim-shNC. We identified 38 differentially expressed lncRNAs between the two groups (Figure 6B), among which 21 were upregulated and 17 were downregulated in the PTEC-Bim-shRNA cells.

Subsequently, we chose the top 10 differential lncRNAs in the PTEC-Bim-shRNA vs control PTECs, and the 6 overlaps in the comparison of PTEC-Bim-shRNA vs PTEC-Bim-shNC for the similar influence between control PTECs and PTEC-Bim-shNC. As shown in Figure 6C, a total of 14 lncRNAs were chosen for further validation. To validate the microarray data, RT-PCR was used to analyze the expression of 14 lncRNAs. Figure 6D shows that expression levels of lncRNA NONHSAT179542.1, NONHSAT040129.2, and NONHSAT203700.1 by RT-PCR were consistent with the microarray data and were upregulated in the PTEC-Bim-shRNA cells, whereas NOHSAT253617.1, XR_929282.2, and other lncRNAs lost significance.

### Functional annotation of differentially expressed lncRNAs

**Figure 7:**
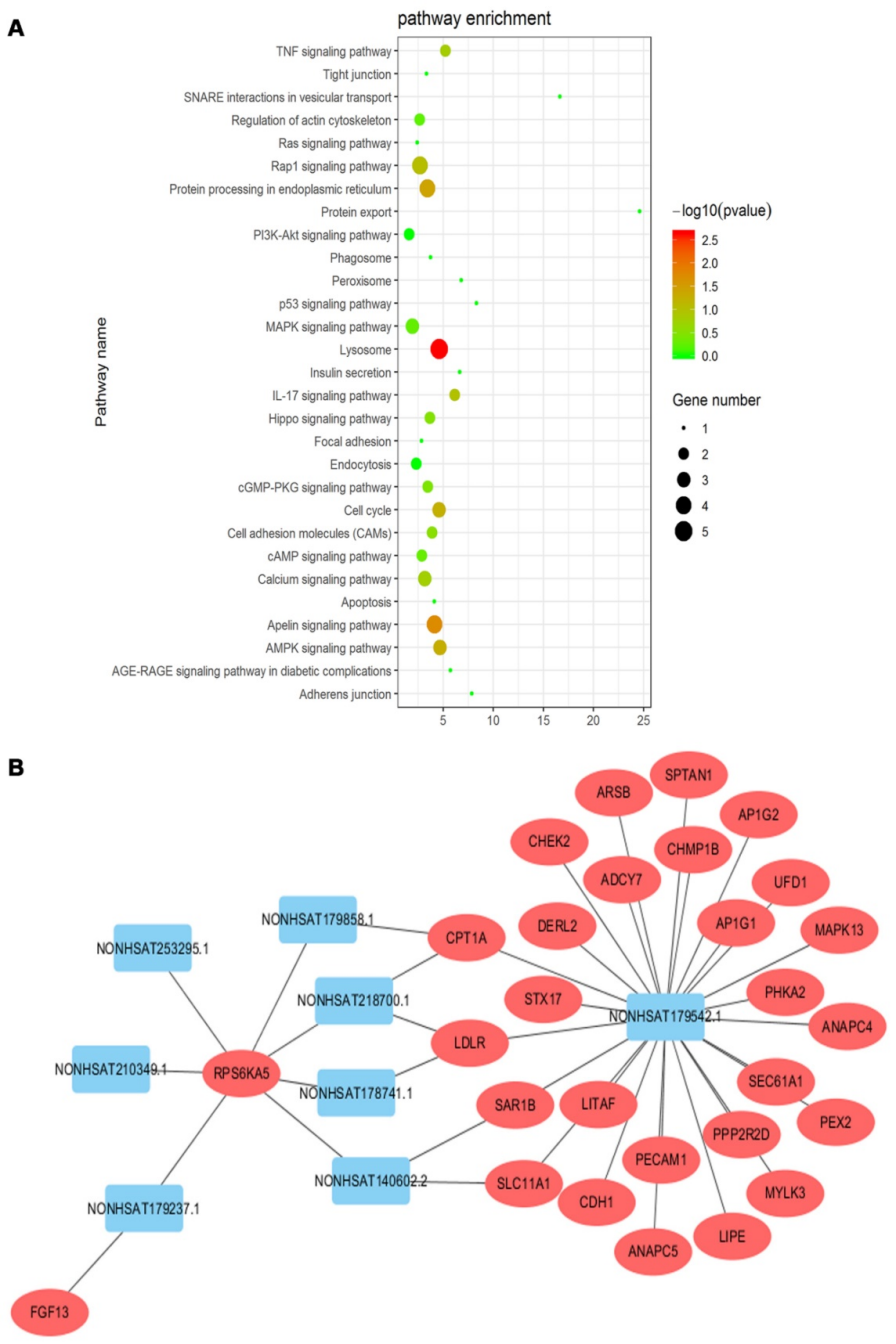
(**A**) KEGG pathway enrichment analysis was conducted, and 29 pathways correlated with cytoskeleton organization were selected. (**B**) Target gene prediction of lncRNAs by cis/trans was conducted. Twenty-eight targeted genes involved in the cytoskeletal pathway were identified, and lncRNA NONHSAT179542.1 was the most central lncRNA in the network.

The KEGG pathway analyses of differentially expressed mRNAs could provide a clue about the PC cytoskeletal disorganization. We screened 29 pathways that were reported to be associated with cytoskeletal disarrangement, and pathway enrichment is displayed in Figure 7A. Combined with data from the KEGG pathway and target gene prediction, a target gene network, involving 8 lncRNAs including NONHSAT179542.1 and 28 targeted genes, was constructed to illustrate the critical association of lncRNAs with cytoskeletal disorganization (Figure 7B). NONHSAT179542.1 was considered to be the most central lncRNA in the network as its 26 targeted genes were involved with cytoskeleton-related pathway enrichment.

### NONHSAT179542.1 downregulation in PCs participated in Bim-mediated cytoskeletal reorganization

**Figure 8:**
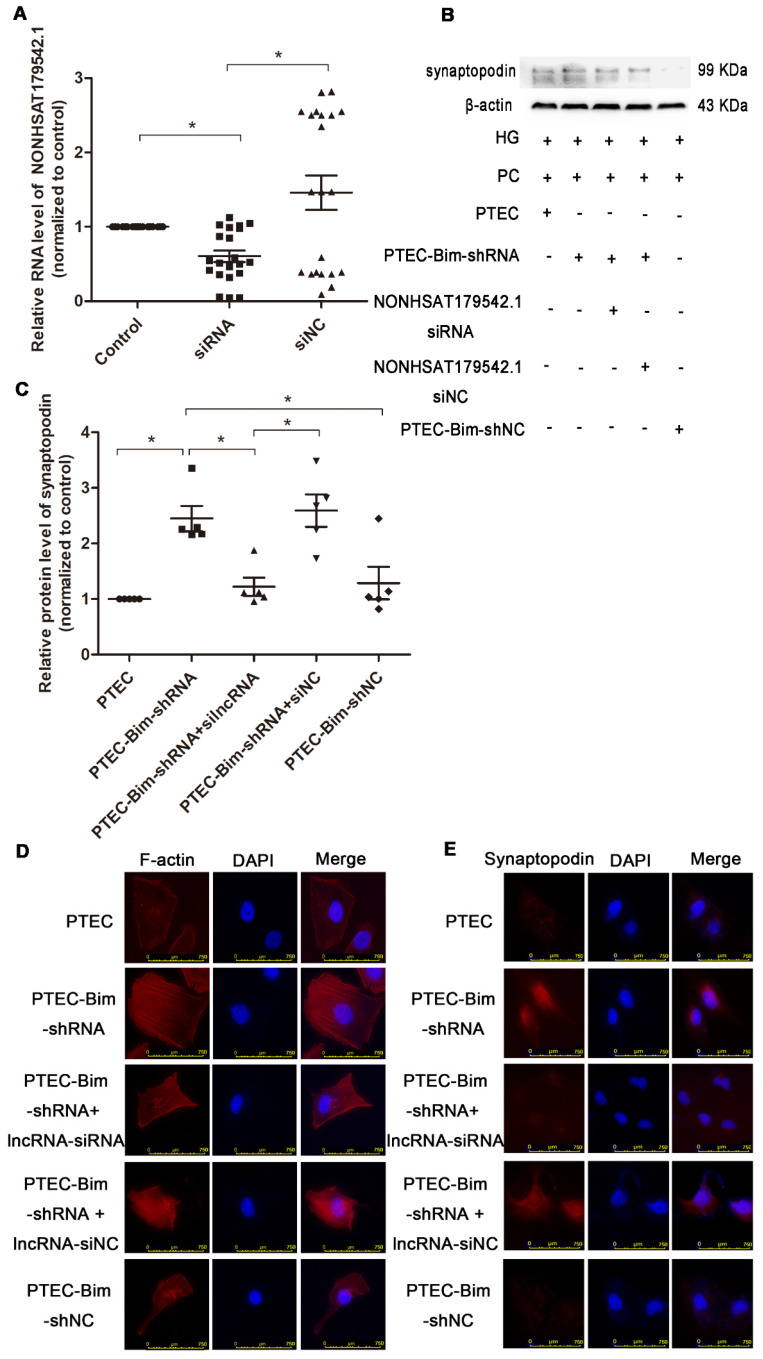
** Validation of lncRNA knock-down efficiency and effects of lncRNA NONHSAT179542.1 alteration on the PC cytoskeletal organization.** (**A**) NONHSAT179542.1 knock-down efficiency was determined by RT-PCR analysis and NONHSAT179542.1 expression was suppressed after transfection with NONHSAT179542.1 siRNA. (**B**) Western blotting showed increased synaptopodin level in PCs by suppression of Bim was inhibited after transfection with NONHSAT179542.1 siRNA compared with negative control (siNC). (**C**) Quantification of synaptopodin protein expression in PCs after the regulation of Bim and NONHSAT179542.1 by Western blotting. (**D**) IF staining showed that suppression of NONHSAT179542.1 markedly disrupted the cytoskeletal organization of PCs by coculturing with PTEC-Bim-shRNA in HG. Original magnification: 400×. (**E**) IF staining showed that inhibition of NONHSAT179542.1 resulted in low expression of synaptopodin, compared with the negative control. Original magnification: 400×. Data are mean ± S.E.M. *P < 0.05.

To investigate the role of NONHSAT179542.1 in cocultured PC cytoskeleton, NONHSAT179542.1 siRNA in PCs was established. The expression of NONHSAT179542.1 was significantly downregulated, indicating successful suppression of NONHSAT179542.1 in PCs (Figure 8A). Subsequently, various coculture treatments were conducted. Western blotting data showed that suppression of NONHSAT179542.1 in PCs decreased synaptopodin level compared with PCs cocultured with PTEC-Bim-shRNA (Figure 8B-C). IF staining for F-actin also showed that downregulation of NONHSAT179542.1 in PCs markedly suppressed cytoskeletal organization, which was also achieved by inhibition of Bim in PTECs (Figure 8D). IF staining of cytoskeletal protein synaptopodin exhibited results similar to F-actin staining (Figure 8E). Thus, these data suggested that downregulated NONHSAT179542.1 participated in PC cytoskeletal reorganization.

### MICAL2 identified as the downstream target gene of NONHSAT179542.1 in PCs

**Figure 9:**
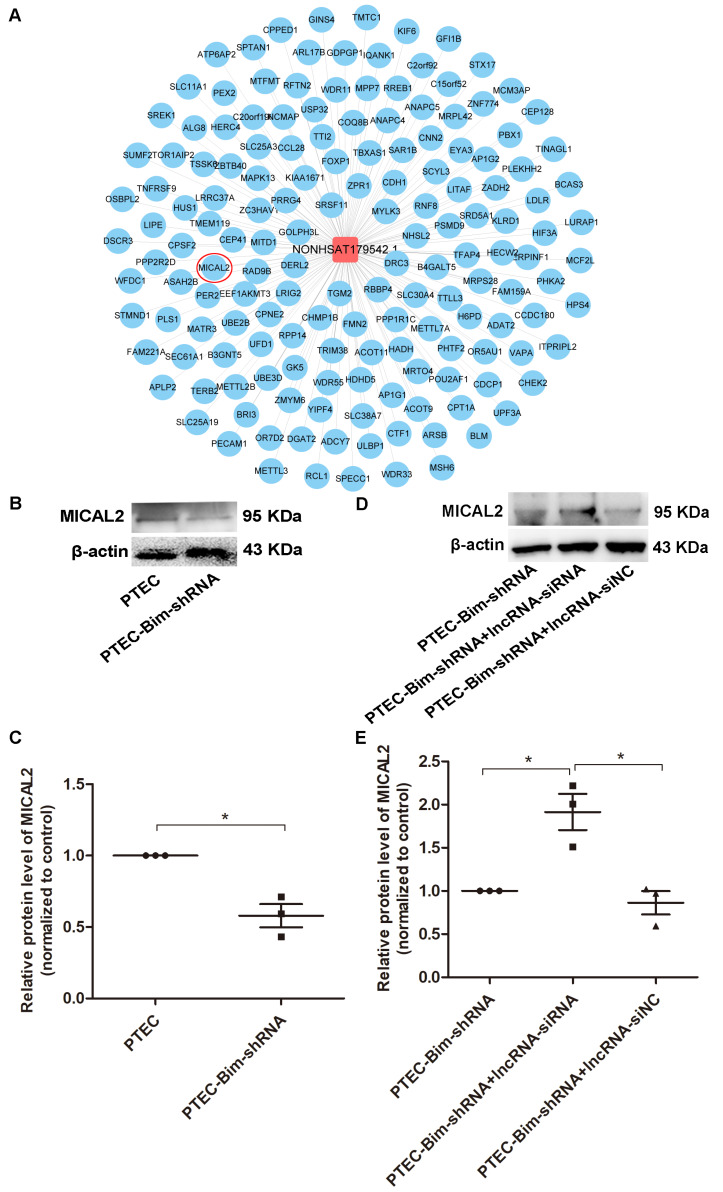
** Validation of MICAL2 as the downstream target of lncRNA NONHSAT179542.1.** (**A**) LncRNA NONHSAT179542.1 and its target genes by cis/trans target gene prediction. (**B**) Western blotting showed decreased MICAL2 level in PCs after coculturing with PTEC-Bim-shRNA compared with control PTECs. (**C**) Quantification of MICAL2 protein expression in PCs after coculturing with PTEC-Bim-shRNA and control PTECs by Western blotting. (**D**) Western blotting showed that after coculturing with PTEC-Bim-shRNA, inhibition of NONHSAT179542.1 upregulated MICAL2 expression in PCs compared with the negative control (siNC). (**E**) Quantification of MICAL2 protein expression in PCs after transfection with NONHSAT179542.1 siRNA and siNC by Western blotting. Data are mean ± S.E.M. *P < 0.05.

We searched for the key downstream target genes of NONHSAT179542.1 using cis/trans target gene prediction. The analysis identified 218 genes as potential targets of NONHSAT179542.1 (Figure 9A). Out of these 218 genes, only a single gene, MICAL2, was present among the differentially downregulated genes identified by microarray analysis in the PCs after coculturing with PTEC-Bim-shRNA compared with control PTECs. Furthermore, Western blotting results showed that compared to control PTECs; the expression of MICAL2 in PCs was decreased when cocultured with PTEC-Bim-shRNA (*P* < 0.05, Figure 9B-C). MICAL2 protein expression was further examined after NONHSAT179542.1 expression was downregulated in PCs and was found to significantly increase compared with the siNC treatment (*P* < 0.05, Figure 9D-E), indicating that MICAL2 was targeted by NONHSAT179542.1.

## Discussion

Current therapeutic strategies to debilitate DN mainly consist of intensive glycemic control and antihypertensive and antiproteinuric measures 31,32. However, these conventional therapies are suboptimal 31, and new therapies to treat and delay the progression of DN are imperative. The focus is also on oxidative stress, inflammation, and fibrosis involved in the pathogenesis of DN 33-35. Our previous finding revealed that HG-induced overexpression of Bim modulated apoptosis of PTECs, which further contributed to the development of early DN 22. Furthermore, Bim was reported to increase cytokine production 26, indicating that overexpressed Bim could potentially influence the peripheral cells of PTECs.

DN is a glomerular disease with impaired GFB 36. The defect in the GFB with increased glomerular permeability usually results in proteinuria 37, which is the main characteristic feature of DN 38. GFB is composed of various cell types, and glomerular visceral epithelial cells, also known as visceral PCs, are critical for the overall functioning of the glomerulus. Damage to visceral PCs is typically implicated in the pathogenesis of progressive development of proteinuria 39,40. PCs' function depends on their specific actin cytoskeletal system, and the cytoskeleton-related proteins which are normally expressed in the cells 41. Among several pathological mechanisms, derangement of the actin cytoskeleton was reported to be a critical feature of impaired PCs 42,43. Consistent with this observation, our current study indicated that cytoskeletal alterations in PCs, manifested by F-actin rearrangement and decreased synaptopodin expression were induced by HG. We observed cytoskeletal abnormalities after HG exposure for 48 h. However, a nonsignificant change in the PC cytoskeleton was detected after a longer period of HG treatment (72 h). This could be because the cytoskeletal disorder of PCs was an early event in DN 44,45, and by 72 h of protracted time period 46, PCs were extensively damaged.

Recent studies have shown that tubular functional defects preceded the onset of albuminuria 47-49. Herein, we investigated the role of Bim in mediating the communication between tubules and PCs. Since the cells must co-exist in the same milieu to explore this possible communication, we established a simple and reliable *in vitro* system in which PCs were grown in a cell culture well and PTECs were suspended over the PC monolayer on a permeable membrane 50. Independent manipulations of PTECs were feasible in subsequent cocultures for exploring their effect on the PCs.

Our data showed that the increased expression of Bim caused by HG in PTECs exacerbated synaptopodin reduction and cytoskeletal rearrangement in PCs, indicating that HG-induced alteration of Bim in PTECs could promote a disordered cytoskeletal organization in PCs. Furthermore, Bim inhibition alleviated the cytoskeletal disorder in PCs, which further confirmed the potential role of Bim in PTECs to induce cytoskeletal disorganization in the peripheral PCs. These findings provided preliminary evidence of the crosstalk between PTECs and PCs in DN.

Physiological homeostasis depends on the tight control of the balance between cell-cell interactions. For kidney cells to function as an integrated filtration unit, cell-cell communication or crosstalk executes biological functions. Particularly, the proximity of PCs and PTECs within the nephron suggests that their intercellular communication is essential. Several studies revealed a potential correlation and possible communication between PCs and PTECs. Advanced glycation end products (AGE) have been shown to correlate biomarkers of PC damage with proximal tubule dysfunction, raising the possibility of an AGE-mediated impact on both glomeruli and proximal tubules 51-53. Hasegawa et al. reported that in the early stage of DN, PTECs exhibited abnormal energy metabolism, and then affected the glomerular PCs by releasing nicotinamide mononucleotide, leading to the abnormal structure and function of PCs. Based upon these observations, the correlation between PTECs and PCs *in vivo* was proposed 54,55. Our current study elucidated that the alteration of Bim in PTECs caused cytoskeletal changes in PCs, providing a new perspective for the influence of cell-cell communication in DN development.

Besides pro-apoptosis, the additional regulatory role of Bim in inducing cytokine production via activation of the nuclear factor of activated T cells (NFAT) was clarified 26. NFAT, a group of transcription factors ubiquitously expressed in mammalian tissues, plays a critical role in orchestrating the intricate cellular interactions 56. The NFAT family consists of five subtypes, NFAT1-5. The sequences of five subtypes have high homology but exert various biological functions by recognizing different calcium signals 57.

In the present study, we analyzed the expression changes of all five NFAT family subtypes in PTECs, and identified NFAT2 as a potential downstream target of Bim. NFAT2 functions as a transcription factor and is involved in gene regulation by binding to specific gene promoters, requiring its translocation to the nucleus. We observed increased nuclear levels of NFAT2 in HG-treated PTECs that overexpressed Bim, which was highly suggestive of nuclear translocation of NFAT2. On the contrary, low levels of Bim led to reduced nuclear translocation of NFAT2, again indicating Bim-induced NFAT2 nuclear translocation. These findings indicated that the downstream target of Bim, NFAT2, might be important in mediating the cytoskeletal disorganization in PCs. Further studies revealed that inhibition of NFAT2 abrogated cytoskeletal disruption in PCs after coculturing with PTECs, while up-regulation of NFAT2 induced synaptopodin reduction and cytoskeletal rearrangement in PCs despite suppression of Bim. These results revealed NFAT2's role as a potential target of Bim involved in the cytoskeletal damage of PCs.

Previously, Ca^2+^-dependent remodeling of the actin cytoskeleton in PCs was shown to be essential to counteract mechanical forces in response to mechanical load 58-60. Faul et al. have shown that cyclosporin A, a calcineurin inhibitor, prevented synaptopodin degradation *in vitro* and mice resistant to synaptopodin degradation were protected from proteinuria *in vivo* 61. Consistent with these findings, we have shown that inhibition of NFAT2 in PTECs achieved similar changes in PCs, including upregulation of synaptopodin and prevention of F-actin cytoskeletal disruption. Thus, we have uncovered a previously unrecognized role of Bim/NFAT2 in PTECs in regulating PCs' cytoskeletal organization.

An important role of hyperglycemia in abnormal activation of T cells has been described previously 62. T cell receptor (TCR), a unique antigen-recognizing receptor on T cells, was shown to be necessary for regulating the activation of Bim/NFAT signaling pathway 26,63. Once TCR was activated, Bim could mediate the activation of autoreactive T cells by promoting phosphorylation of NFAT 26. Intriguingly, PTECs were recognized as the nephron segment most susceptible to T cells and activated infiltrating T cells played a crucial role in nephritic inflammation 64, further promoting apoptosis of PTECs 65. Combined with the fact that DN patients often suffered from hyperglycemia, which potentially activated TCR 66, we inferred TCR acted as the upstream of Bim/NFAT2 pathway in PTECs, which regulated the cytoskeletal organization in PCs.

To further explore the molecular mechanisms involved in the cytoskeletal disruption in PCs, we performed lncRNA microarray analysis of PCs cocultured with PTECs after the regulation of Bim. LncRNAs, with sequence length greater than 200 nucleotides, have been suggested to regulate the gene expression during normal development and diseases, including DM and other complex disorders 67-73. The number of reported lncRNAs is up to 70,000, far more than mRNA 68. It has been shown that lncRNA played a critical role in transforming growth factor-β/Smad3-mediated renal inflammation and fibrosis 71. Also, lncRNA was functionally vital in modulating renal response to hyperglycemia and the progression of DN 72. Besides, lncRNA Tug1 overexpression in PCs improved diabetes-induced chronic kidney disease 73. Although there is some evidence correlating the occurrence of DN and lncRNAs, inadequate information is available about the role of lncRNAs in Bim-mediated cytoskeletal damage in PCs.

To address this issue, we used Sino-human lncRNA array V3.0 to detect the differentially expressed lncRNA profile in PCs after coculturing with PTECs. Several differentially expressed lncRNAs were selected for further verification by RT-PCR, identifying NONHSAT179542.1, NONHSAT040129.2, and NONHSAT203700.1 for subsequent studies. Based on KEGG functional annotation data and target gene prediction of lncRNA microarray analysis of PCs, NONHSAT179542.1 was chosen for further analysis. Functional studies showed that inhibition of NONHSAT179542.1 in PCs markedly exacerbated the cytoskeletal damage which was alleviated by suppression of Bim in PTECs, indicating NONHSAT179542.1's role in Bim-mediated PC cytoskeletal reorganization in DN. Downstream mechanism of NONHSAT179542.1 was further explored and 218 genes were identified as the potential targets of NONHSAT179542.1 by cis/trans target gene prediction. By microarray data analysis and the NONHSAT179542.1 regulation study, MICAL2, a flavoenzyme that binds to F-actin and triggers its depolymerization through redox modification 74,75, was recognized as the key target gene. Thus, our findings identified the potential role of NONHSAT179542.1/MICAL2 in Bim-mediated cytoskeletal disorganization in PCs. Our study has helped enrich the molecular understanding of the Bim-mediated regulatory network connecting tubular and PC interactions in the pathogenesis of DN. Further investigations are required to clarify the regulation of NONHSAT179542.1/MICAL2 in the Bim-mediated cytoskeletal disorder in PCs to realize the significant clinical potential of the molecular details of DN development elucidated by our studies.

## Conclusions

In this study, we have provided evidence that HG-induced Bim overexpression in PTECs exacerbated PC cytoskeletal disruption by targeting NFAT2 and the key downstream targets, lncRNA NONHSAT179542.1/MICAL2 were implicated in it. Our data clarified insightful molecular mechanisms of tubule-PC crosstalk as a potential approach in DN treatment.

## Supplementary Material

Supplementary figures and tables.

## Abbreviations

DN: diabetic nephropathy
PTECs: proximal tubular epithelial cells
PC: podocyte
HG: high glucose
F-actin: filamentous actin
DM: diabetes mellitus
Bim: B cell lymphoma 2-interacting mediator
GFB: glomerular filtration barrier
FBS: fetal bovine serum
NG: normal glucose
PET: Polyester
Syn: synaptopodin
KEGG: Kyoto Encyclopedia of Genes and Genomes
NFAT: nuclear factor of activated T cells
