# Supplementary material for: Renal tubular Bim mediates the tubule-podocyte crosstalk via NFAT2 to induce podocyte cytoskeletal dysfunction
Source: Theranostics. 2020 May 22;10(15):6806–24. doi: 10.7150/thno.43145 (PMC7295056; doi:10.7150/thno.43145)
Supplement: Supplementary file 1 — Supplementary figures and tables. [file thnov10p6806s1.pdf]

Supplemental Material

Table S1. Primers for RT-PCR

Primer name	Forward (5'-3')	Reverse (5'-3')
Bim	ATTACCAAGCAGCCGAAGAC	TCCGCAAAGAACCTGTCAAT
NFATc1	GATGCCAAGCACCAGCTTTC	CCATAGTGTTCTTCCTCCGCT
NFATc2	CAGAGAGAGGCTGCGTTCAG	TGCATTCGGCTCTTCTTGGT
NFATc3	ACAGTTTGTGTGATCCAGCG	ACCTGATGGCTGGTGTTCC
NFATc4	GGGGATTGGGGGAAGAACTG	GATAGGTGCAGCGCCATAGG
NFATc5	TGTTGTTGCTGCTGATGCTTCTT	TTACTTACCCCCACGGCTGA
NONHSAT253617.1	CCCATTGAACTGACCTAATCTCTTA	GGCAGTTCACAGATAGTCCAGCA
XR_929282.2	ACGATTTCAGACAAGCAGGCA	ACTTTGACCTTGGGCTCGG
NONHSAT179542.1	AAGAAAGGAAAGATTGTGGCCC	CCACAAGACTGTTGGCTTTCTACT
NONHSAT251382.1	CCTTCCTCTGCCACTGATAATAGT	GGTCCAAAACGCCAAAAATG
XR_934942.2	TCTGATGCTTATTGCCAACTTG	CCAATTTGAGAAGAGATTTGTGTCC
NONHSAT220341.1	GGTTTTGTTCGCTTGCACTAA	GACGGACAGTTAGGTTGAGGAAC
ENST00000542022	TAGAAGCTCAGCCAAGAGCTCC	AACAAACTCCAGAACAGCAGAAAG
NONHSAT040129.2	GTAACGTTGGGTCTGTAAATGTGT	TTTGAGCCAGGTGATCTGCTC
NONHSAT227535.1	TCTACAAAAAGCTGTGTTTCTCCC	CTGGATGAGAATATTGCACCACA
NONHSAT179237.1	TAGGGCAAATTTGGAAAAGCAG	GGCCTTTCAGAAAAACATCTCCT
NONHSAT203700.1	CTAAAGCTGCCTCAGACACTTGACT	CCTTTATTGTGAAGCCTACATTTCC
NONHSAT243847.1	AGCTGGCAACTTGCAAATATATC	CTCAATACCATGGACCTGAAGC
NONHSAT179858.1	GAATGACTTACTTCTCTGGTTTGCT	GACAGGAGTGGGACTTGGCT
NONHSAT040287.2	ACTGTGTCCAAAGACAAGGCTG	GGCTTTGAAGATGCTTGTTTCA

β-actin	TGACGTGGACATCCGCAAAG	CTGGAAGGTGGACAGCGAGGT

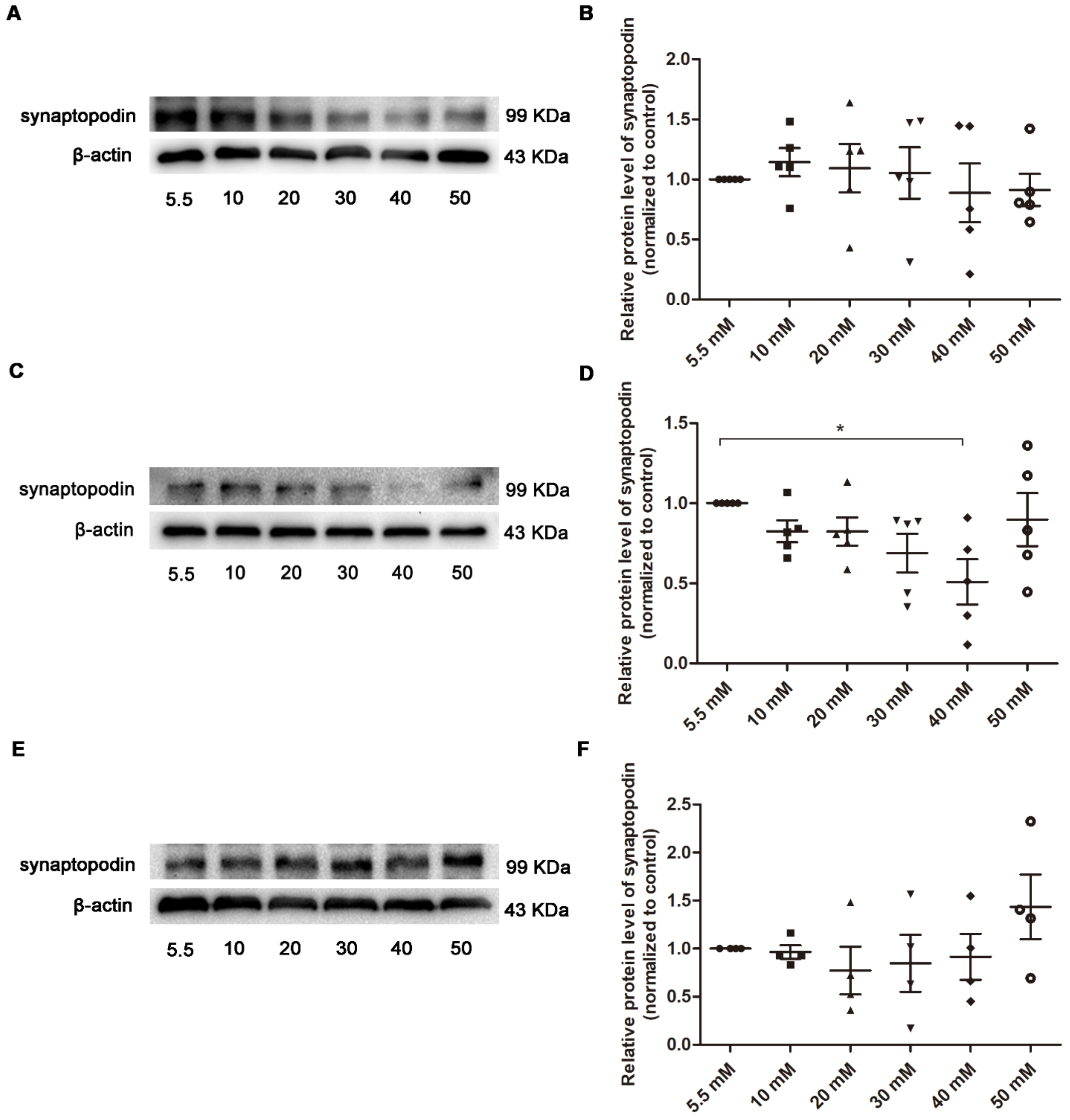

Figure S1. Effect of various glucose concentration treatment for 24 h, 48 h, and 72 h on the synaptopodin level in PCs. (A) Western blotting showed no significant difference in synaptopodin levels in PCs when treated with different HG concentrations for 24 h compared with NG. (B)

Quantification of synaptopodin protein expression in PCs when treated with different HG concentrations for 24 h. (C) Western blotting demonstrated a significant decrease in the synaptopodin level in PCs when treated with 40 mM HG for 48 h compared with NG. (D) Quantification of synaptopodin protein expression in PCs when treated with different HG concentrations for 48 h. (E) Western blotting showed no significant difference in synaptopodin levels in PCs when treated with different HG concentrations for 72 h compared with NG. (F) Quantification of synaptopodin protein expression in PCs when treated with different HG concentrations for 72 h. Data are mean ± S.E.M. *P < 0.05. PCs: podocytes.

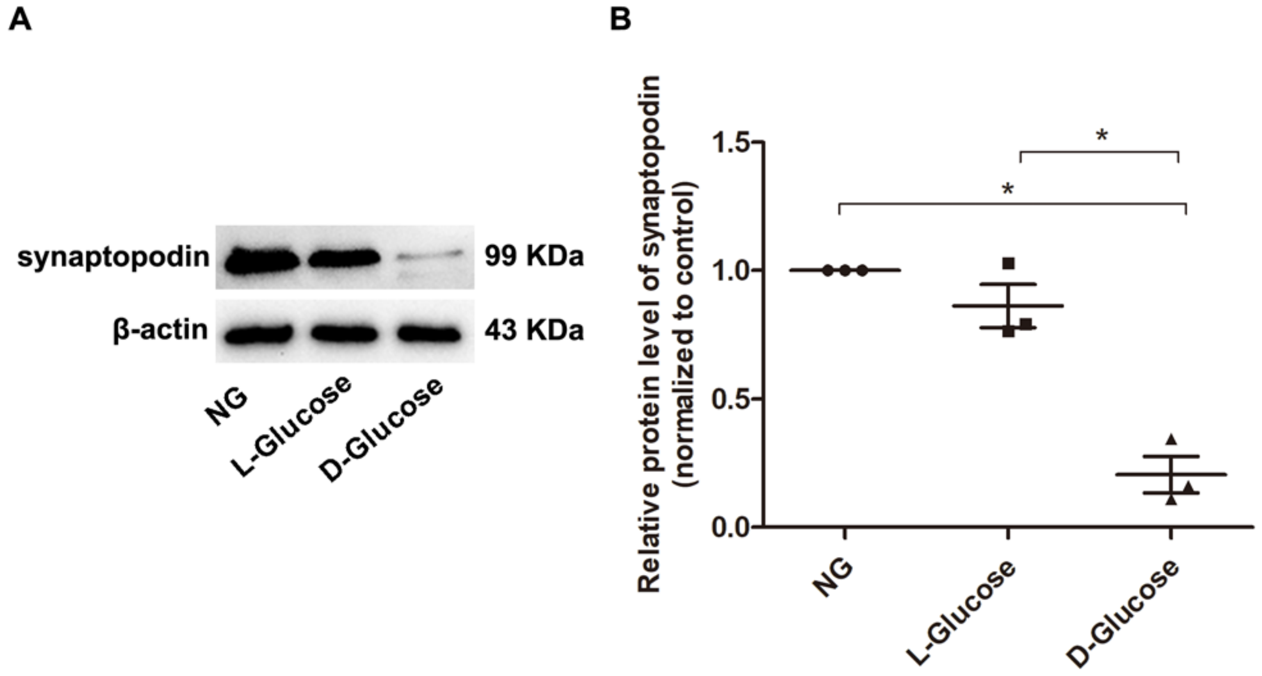

Figure S2. Effect of high D-glucose on synaptopodin level in PCs. (A) Western blotting showed that high D-glucose significantly reduced the expression of synaptopodin in PCs compared with NG. In contrast, with high L-glucose, treatment with high D-glucose reduced synaptopodin expression in PCs. (B) Quantification of synaptopodin protein expression in PCs after various glucose treatments. Data are mean ± S.E.M. *P < 0.05. NG: normal glucose.

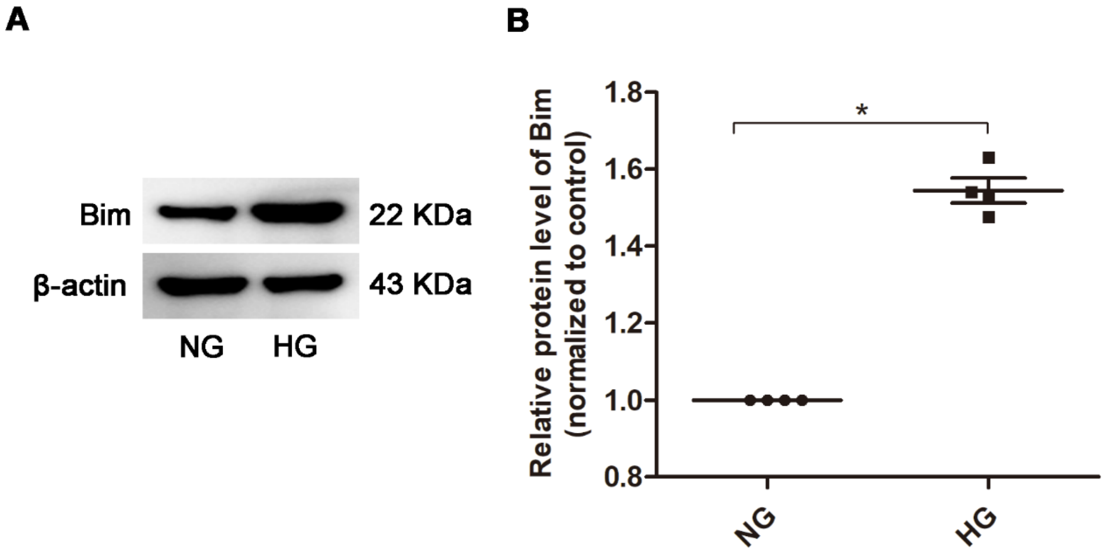

Figure S3. Effect of HG on the Bim level in PTECs. (A) Western blotting showed significantly increased expression of Bim in PTECs in response to HG. (B) Quantification of Bim protein expression in PTECs after HG treatment. Data are mean ± S.E.M. **P* < 0.05. HG: high glucose. PTECs: proximal tubular epithelial cells.

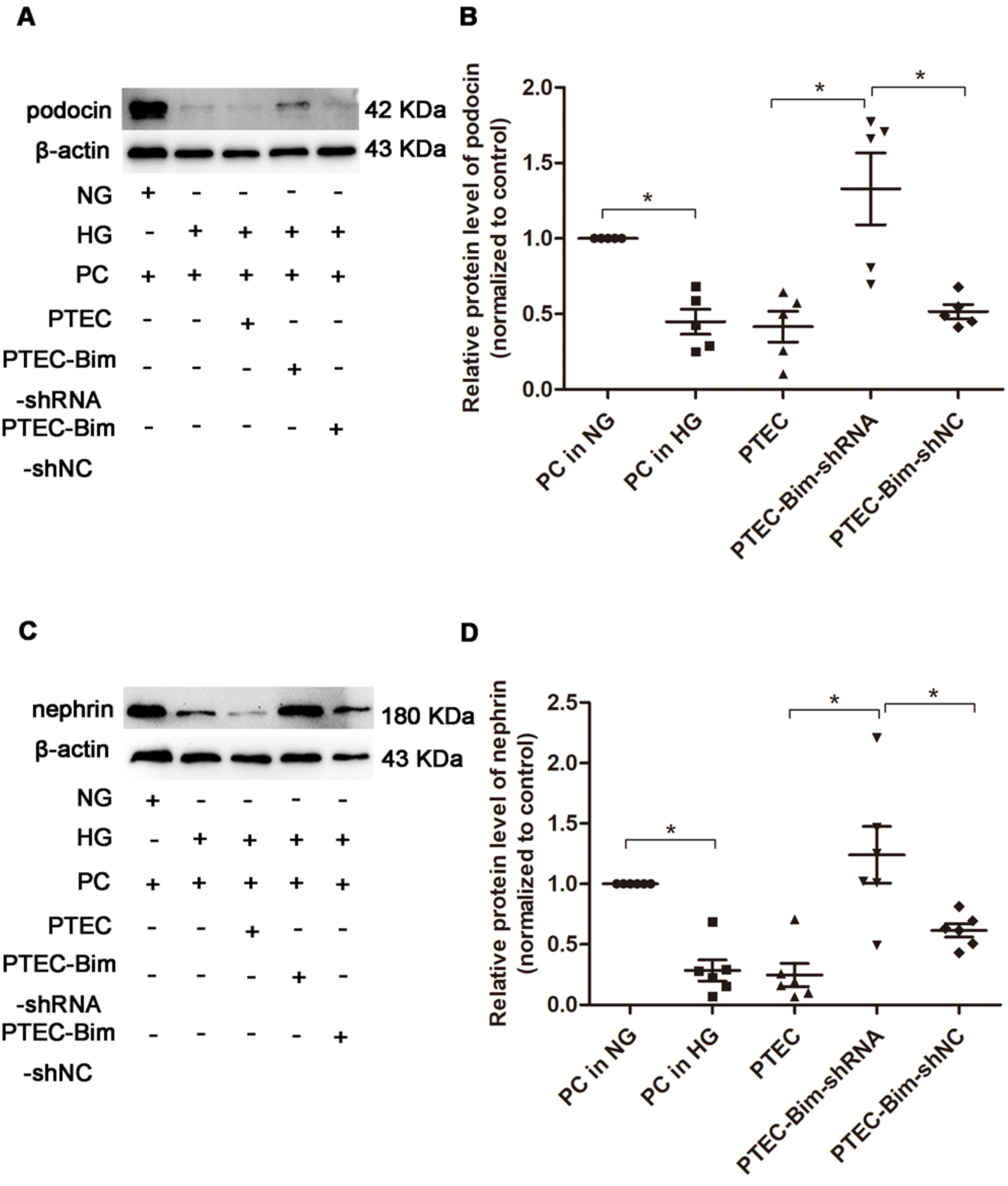

Figure S4. Effect of different treatments on specific proteins in PCs. (A) Western blotting showed HG significantly decreased podocin level compared with the NG. Podocin level was significantly up-regulated in PCs after coculturing with PTEC-Bim-shRNA compared to negative

control (PTEC-Bim-shNC). (B) Quantification of podocin protein expression in PCs was detected by Western blotting. (C) Western blot analysis showed HG significantly decreased nephrin level compared with the NG. Nephrin level was significantly up-regulated in PCs after coculturing with PTEC-Bim-shRNA compared to PTEC-Bim-shNC. (D) Quantification of nephrin protein expression in PCs by Western blotting. Data are the mean ± S.E.M. *$P < 0.05$.

A

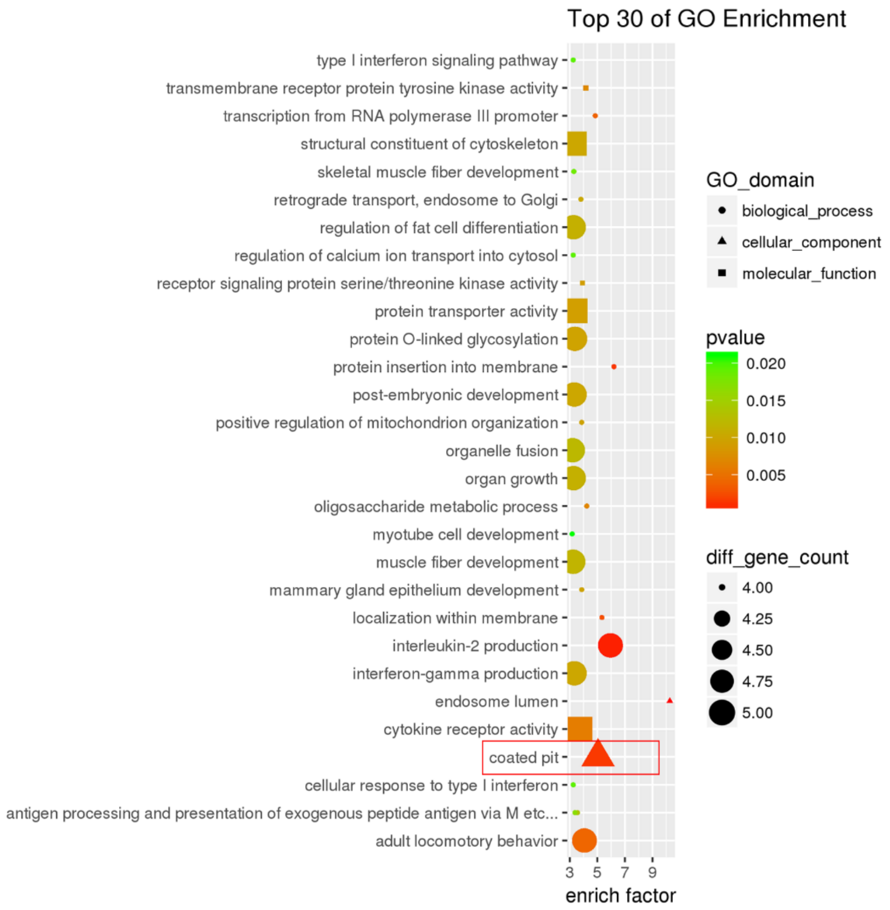
Top 30 of GO Enrichment
type I interferon signaling pathway
transmembrane receptor protein tyrosine kinase activity
transcription from RNA polymerase III promoter
structural constituent of cytoskeleton
skeletal muscle fiber development
retrograde transport, endosome to Golgi
regulation of fat cell differentiation
regulation of calcium ion transport into cytosol
receptor signaling protein serine/threonine kinase activity
protein transporter activity
protein O-linked glycosylation
protein insertion into membrane
post-embryonic development
positive regulation of mitochondrion organization
organelle fusion
organ growth
oligosaccharide metabolic process
myotube cell development
muscle fiber development
mammary gland epithelium development
localization within membrane
interleukin-2 production
interferon-gamma production
endosome lumen
cytokine receptor activity
coated pit
cellular response to type I interferon
antigen processing and presentation of exogenous peptide antigen via M etc...
adult locomotory behavior
3
5
7
9
enrich factor
GO_domain
biological_process
cellular_component
molecular_function
pvalue
0.020
0.015
0.010
0.005
diff_gene_count
4.00
4.25
4.50
4.75
5.00

B

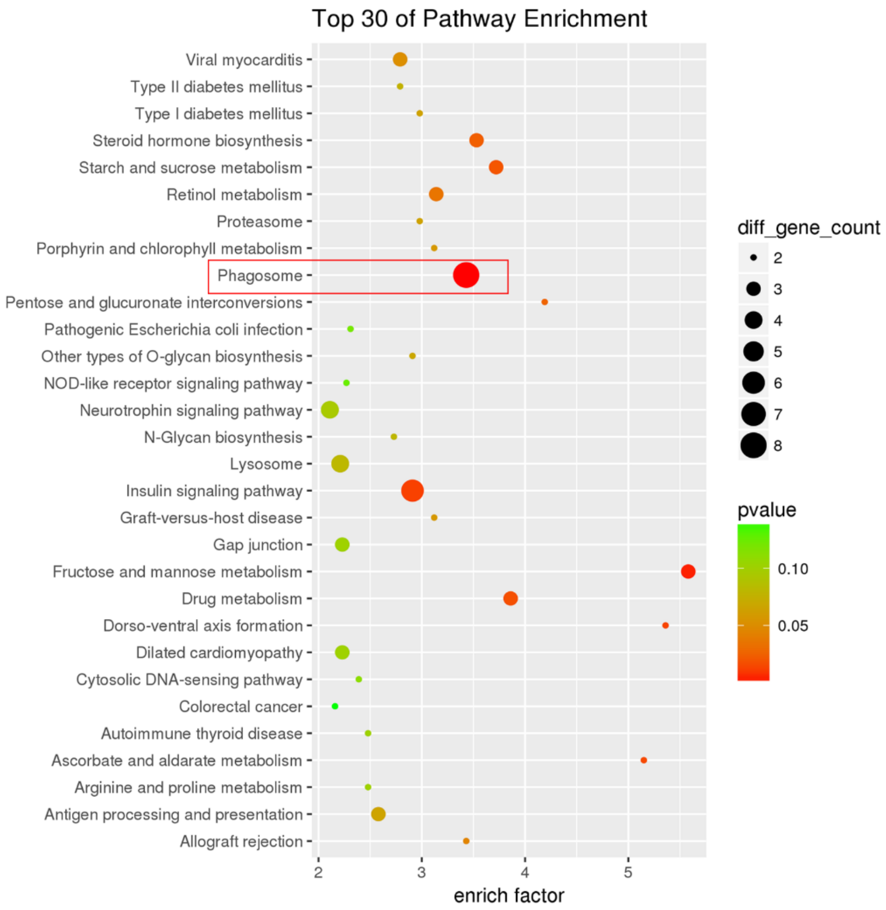
Top 30 of Pathway Enrichment
Viral myocarditis
Type II diabetes mellitus
Type I diabetes mellitus
Steroid hormone biosynthesis
Starch and sucrose metabolism
Retinol metabolism
Proteasome
Porphyrin and chlorophyll metabolism
Phagosome
Pentose and glucuronate interconversions
Pathogenic Escherichia coli infection
Other types of O-glycan biosynthesis
NOD-like receptor signaling pathway
Neurotrophin signaling pathway
N-Glycan biosynthesis
Lysosome
Insulin signaling pathway
Graft-versus-host disease
Gap junction
Fructose and mannose metabolism
Drug metabolism
Dorso-ventral axis formation
Dilated cardiomyopathy
Cytosolic DNA-sensing pathway
Colorectal cancer
Autoimmune thyroid disease
Ascorbate and aldarate metabolism
Arginine and proline metabolism
Antigen processing and presentation
Allograft rejection
2
3
4
5
enrich factor
diff_gene_count
2
3
4
5
6
7
8
pvalue
0.10
0.05

Figure S5. GO term and KEGG pathway enrichment analysis of differentially expressed lncRNA in PCs. (A) GO term analysis showed that differentially expressed lncRNAs in PCs were enriched in coated-pit. (B) KEGG pathway enrichment analysis indicated that the differentially expressed lncRNAs in PCs were enriched in phagosome. lncRNA: long noncoding RNA.
